# Intradural extramedullary meningeal melanocytoma: a case report and literature review

**DOI:** 10.1093/jscr/rjad002

**Published:** 2023-01-26

**Authors:** Rabeeia Parwez, Razna Ahmed, Arsalan Baig, Fernanda Ruiz, Asfand Baig Mirza, Ahmed-Ramadan Sadek, Babak Arvin, Anjum Qureshi

**Affiliations:** Department of Neurosurgery, Queens Hospital Romford, London, UK; GKT School of Medical Education, King’s College London, London, UK; Department of Neurosurgery, Queens Hospital Romford, London, UK; Department of Neuropathology, UCL Institute of Neurology, London, UK; Department of Neurosurgery, Queens Hospital Romford, London, UK; Department of Neurosurgery, Queens Hospital Romford, London, UK; Department of Neurosurgery, Queens Hospital Romford, London, UK; Department of Neurosurgery, Queens Hospital Romford, London, UK

**Keywords:** thoracic melanocytoma, extradural intramedullary lesion, meningeal melanocytoma, spinal melanocytoma

## Abstract

Primary meningeal melanocytomas are extremely rare, benign tumours arising from the leptomeninges. While they are considered to be benign lesions, there is potential for their growth and transformation into malignant melanomas. They are commonly found in the cervical spine, with a decreased incidence in the thoracic and lumbar regions. We present a case report of a 56-year-old man who presented to our unit with a 4-month history of lower limb weakness and a sensory level at T6. Magnetic resonance imaging shows an intradural extramedullary tumour. The patient underwent a thoracic debulking of the lesion with neurophysiological monitoring. Histopathology confirmed the diagnosis of melanocytoma of meningeal origin, with a low mitotic count. Our patient recovered well post-operatively with no complications. Surgical resection is an effective method to manage this tumour; however, adjuvant radiotherapy is advised due to the risk of recurrence and malignant transformation.

## INTRODUCTION

Meningeal melanocytomas are extremely rare benign tumours arising from the leptomeninges; 1 case presents per 10 million people every year [1]. They were first described in 1972 by Limas and Tio as primary melanotic tumours of the leptomeninges [2]. They typically behave as slow-growing, well-circumscribed tumours in the spinal cord, however, can also be found in the brain [3]. They are most commonly located in the upper cervical region of the spinal cord due to the greater concentration of melanocytes [1]. These tumours can be both intra-/extradural and intra-/extramedullary [3].

In this paper, we present a 56-year-old male with neurological decline secondary to this rare, intradural extramedullary tumour.

## CASE PRESENTATION

A 56-year-old male presented with a 2-month history of recurrent falls and a past medical history of diabetes mellitus, hypertension and primary hyperparathyroidism. He fell down a flight of stairs 10 weeks prior to admission, resulting in a back injury and a significant decline in the power of his legs.

**Figure 1:**
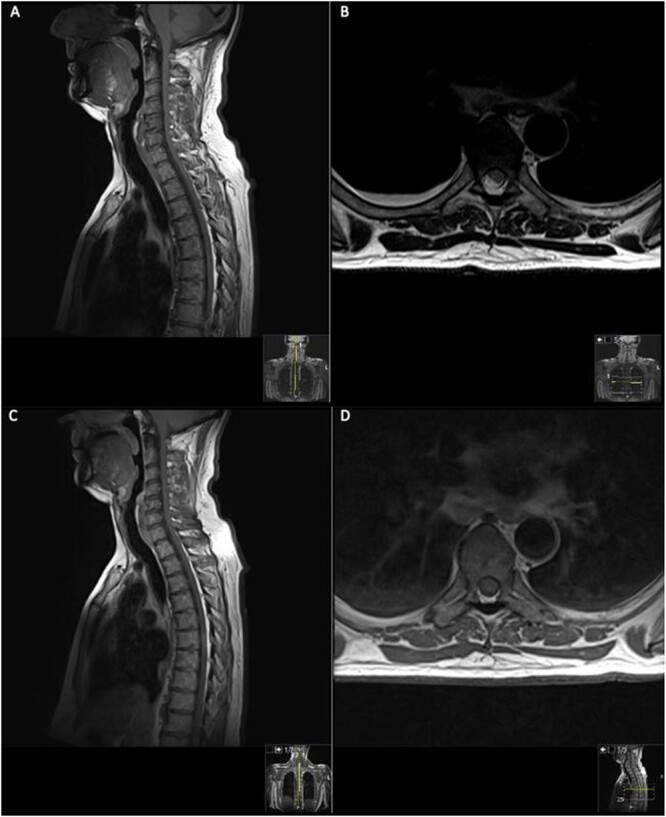
MRI on initial presentation; T1 (**A**) sagittal view sowing mild high signal changes alongside a T2 sequence transverse view (**B**) which shows the intermediate signal change; when compared with post-contrast T1 sagittal and transverse imaging (**C**) and (**D**), showing there is no significant enhancement post-contrast. MRI revealed an intramedullary spinal cord lesion at the level of T6 measuring 24 mm in craniocaudal dimension.

On presentation to A&E, his lower limb power was MRC 4/5 in the right leg and 5/5 in the left leg. Muscle power, sensation and reflexes in the upper limb were intact. He had upgoing plantars and normal lower limb reflexes, except for hyporeflexia in the right ankle reflex. Pinprick sensation was intact until T6 level, however, perianal light touch and pinprick sensation were absent. Urinary function and anal tone remained intact. An magnetic resonance imaging (MRI) spine was requested and discussed by the multidisciplinary team (MDT) (Fig. 1).

**Figure 2:**
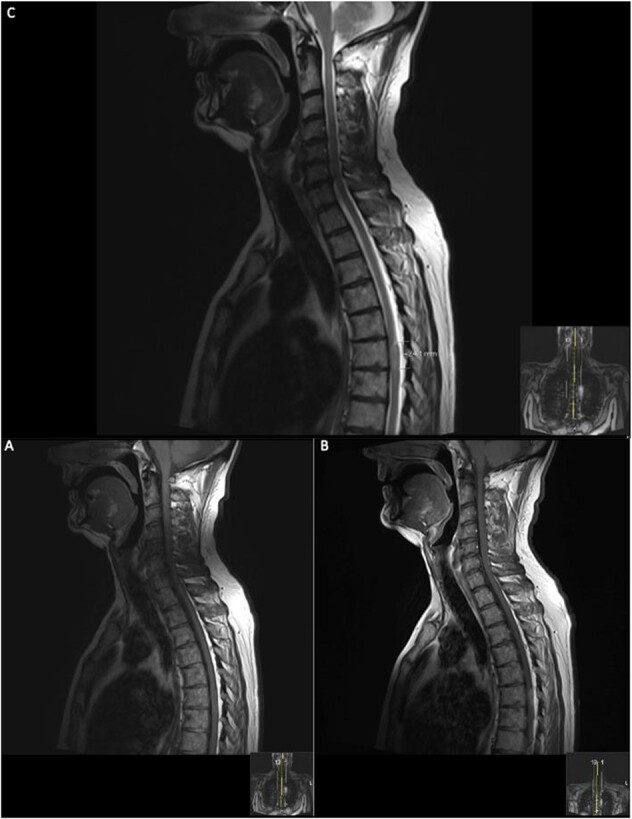
Repeat contrasted MRI whole spine; demonstrates findings consistent with spinal cord meningioma at the level of T6; this can be clearly visualized by comparing the T1 sagittal pre-contrast (**A**) with a T1 sagittal post-contrast (**B**), where a clearly demarcated lesion can be seen at the level of T6; this is supported further by the sagittal T2 image (**C**) shown where an ~24 mm lesion can be seen at T6.

The patient was started on methylprednisolone (1 g/day) for 3 days under the neurology team; however, this did not improve his neurological status, and subsequently, his neurology progressively worsened to a lower limb weakness to an MRC grade of 1/5 bilaterally with faecal incontinence. A second MRI spine was performed with contrast (Fig. 2).

The patient underwent gross-total resection (GTR) of the intradural extramedullary lesion via a T6 laminectomy with intraoperative neuromonitoring. The monitoring revealed that the MEPs were stable throughout the procedure, with no MEP recording seen in the right lower limb muscles from the beginning of the recording.

### Post-operative outcomes

On Day 1 post-operatively, the patient was able to move his toes, however, had bilateral 0/5 power for ankle dorsiflexion and plantarflexion and 1/5 power for knee extension and flexion. High-dose dexamethasone was started post-operatively.

**Figure 3:**
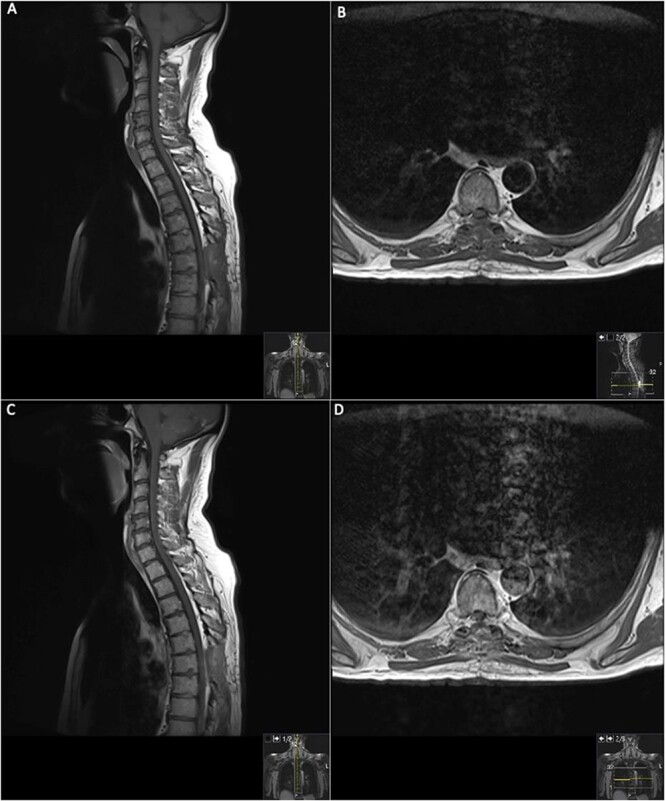
19 days post operative MRI; T1 pre-contrast sagittal (**A**) and T1 pre-contrast axial (**B**) show evidence of surgery at T6. Post-contrast T1 sagittal (**C**) and axial (**D**) show evidence of a small ‘fleck’ of dural enhancement, however, provide no evidence of any definite residual lesion.

On the second day post-operatively, the patient gained 4/5 power in his hips, knees and right ankle and gained 3/5 power in his left ankle. The patient’s urinary catheter was removed 10 days post-operatively. A post-operative MRI was performed after 19 days showing GTR of the lesion (Fig. 3).

The patient recovered well with intensive physiotherapy and prior to discharge had a 5/5 power bilaterally and was mobilizing with a frame.

At the neuro-oncology MDT, a prognostic outcome was not provided due to the rare histopathological diagnosis.

At 3-month follow-up, the patient reported improvement in mobilizing, bladder and bowel control. He also experienced significant improvement in the lower limb power and sensation.

**Figure 4:**
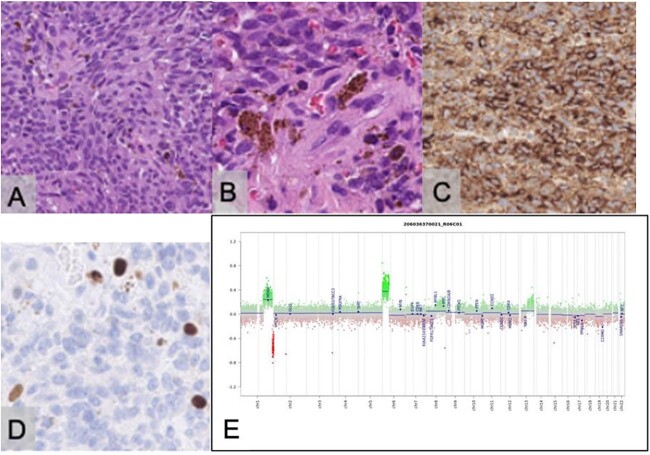
Histopathological examination with haematoxylin and eosin staining reveals a tumour with a storiform pattern (**A**) and widespread melanin pigment deposition (**B**). Immunostaining for HMB45 is positive in the tumour cells (**C**); the Ki67 proliferative index is estimated at 5% (**D**); the scale bar corresponds to 100 μm (A, C) and 50 μm (B, D); copy number assay derived from the methylation array suggests a gain of Chromosome 6p (**E**).

Biopsy demonstrated a well-demarcated tumour with a storiform growth pattern composed of spindle tumour cells with mild nuclear pleomorphism. Intracellular and extracellular deposits of melanin were noted with occasional mitotic figures. Immunostaining for HMB-45 was positive, and ki67 proliferative index was estimated at 5%. Immunostaining for GFAP, STAT6 and EMA was negative. Methylation profiling resulted in the classification of melanocytoma [4] with a calibrated score of 0.99, further supporting the morphological diagnosis of meningeal melanocytoma (Fig. 4).

## DISCUSSION

A preoperative differential of spinal meningeal melanocytomas is challenging due to their non-specific clinical and neurological presentations [5]. Clinical manifestations are usually progressive myelopathy and radiculopathy due to spinal cord and nerve root compression [6] as well as weakness, sensory deficits and progressive pain [5].

Radiologically, they appear as well-defined, extra-axial, isodense-to-hyperdense, homogeneous, contrast-enhancing mass lesions on CT. Their appearance is variable on MRI depending on melanin concentration [5]. MRI hyperintensity on T1-weighted imaging and iso-/hypointensity on T2-weighted imaging caused by melanin help to distinguish meningeal melanocytomas from other meningiomas [6].

Macroscopically, these tumours appear to be black, red or nonpigmented. Microscopically, tumour cells are spindled or oval-shaped and contain melanin in nestlike structures [7]. Cytologic atypia, mitosis, necrosis and microvascular invasion are generally absent and are similarly seen in meningioma. Electron microscopy and immunoperoxidase staining are helpful in differentiating melanocytoma and meningioma [1]. On immunohistochemistry, meningeal melanocytomas are distinctively positive for HMB-45, S-100 protein and vimentin antibodies and are negative for EMA and Leu7 [6].

Complete GTR is the treatment of choice, however, may not always be appropriate due to intraoperative haemorrhage risk [5]. The risk of tumour recurrence is noted even after GTR; therefore, adjuvant radiotherapy is advised [5]. Meningeal melanocytomas are associated with leptomeningeal spread [5] and a high risk of local recurrence [6]. GTR is associated with better survival compared to subtotal resection (STR). Patients undergoing STR benefited from post-operative radiotherapy (dose: 45–55 Gy) [8].

Twenty-three patients were identified, who were predominantly males (*n* = 17). The mean age at which patients first presented was 40.6 years (5–72) [9, 10]. Common presentations include leg weakness (*n* = 8) [6, 7, 9, 11–15], neck pain (*n* = 5) [16–20] and back pain (*n* = 4) [10, 11, 14, 21]. The average duration of symptoms prior to initial presentation was 8.7 months (0.07–36); 43% (*n* = 10) cases were in the cervical region, 43% (*n* = 10) were in the thoracic region, 9% (*n* = 2) were in the sacral region and 4% (*n* = 1) were in the lumbar region of the spinal cord.

In 57% (*n* = 13) of patients, GTR was achieved. The remaining patients (43%, *n* = 10) underwent STR, with the extent of resection determined by the surgeon; 43% (*n* = 10) of patients experienced complete resolution of their symptoms after surgery. Post-operative complications were infrequently reported, however, the most reported was genitourinary dysfunction (*n* = 5) [6, 10, 14, 22, 23]. Four patients were not followed up, but of those that were, the average follow-up period was 16 months (1–96).

In conclusion, despite the rarity of spinal meningeal melanocytoma, timely management can optimize the clinical outcomes for these patients, and more studies are required to gain an understanding of the prognostic outcome of these rare tumours.

## CONFLICT OF INTEREST STATEMENT

None declared.

## FUNDING

No funding was received for this research.

## ETHICAL APPROVAL

All procedures performed in studies involving human participants were in accordance with the ethical standards of the institutional and/or national research committee (name of institute/committee) and with the 1964 Helsinki declaration and its later amendments or comparable ethical standards. Consent was gained from the individual included in this study. For this type of study, formal consent is not required.

## DATA AVAILABILITY

Data is available upon request. Requests to access the dataset should be sent to razna.ahmed@kcl.ac.uk.
